# Lévy Diffusion Under Power-Law Stochastic Resetting

**DOI:** 10.3390/e28010104

**Published:** 2026-01-15

**Authors:** Jianli Liu, Yunyun Li, Fabio Marchesoni

**Affiliations:** 1 MOE Key Laboratory of Advanced Micro-Structured Materials, School of Physics Science and Engineering, Tongji University, Shanghai 200092, China; 2 Dipartimento di Fisica, Università di Camerino, I-62032 Camerino, Italy

**Keywords:** stochastic resetting, Brownian motion, Lévy walk

## Abstract

We investigated the diffusive dynamics of a Lévy walk subject to stochastic resetting through combined numerical and theoretical approaches. Under exponential resetting, the process mean squared displacement (MSD) undergoes a sharp transition from free superdiffusive behavior with exponent $\gamma_{0}$ to a steady-state saturation regime. In contrast, power-law resetting with exponent β exhibits three asymptotic MSD regimes: free superdiffusion for β<1, superdiffusive scaling with a linearly β-decreasing exponent for $1<\beta <\gamma_{0}+1$, and localization characterized by finite steady-state plateaus for $\beta >\gamma_{0}+1$. MSD scaling laws derived via renewal theory-based analysis demonstrate excellent agreement with numerical simulations. These findings offer new insights for optimizing search strategies and controlling transport processes in non-equilibrium environments.

## 1. Introduction

In nature, random motion manifests ubiquitously across microscopic to macroscopic scales, with Lévy walk emerging as a fundamental paradigm alongside Brownian motion. Characterized by heavy-tailed jump length distributions, Lévy walk facilitates efficient long-range exploration through its distinctive combination of localized search and sporadic abrupt relocation [1]. This dynamical strategy has been proposed to explain the performance of certain biological systems [2,3,4], including albatross foraging patterns [2], bacterial chemotaxis [3], and cell migration in vivo [4], and has been theoretically validated as an optimal search mechanism in sparse environments [5,6]. Unlike Brownian motion, whose diffusive behavior is governed by short-range fluctuations, Lévy walk’s power-law statistics enables it to overcome the “curse of dimensionality” in target searches. Lévy walk analysis, though occasionally viewed as an idealized construct [7], has emerged as a central paradigm for characterizing non-equilibrium transport across diverse physical and biological contexts [8].

When searching for a target in a crowd, if one cannot find it within an affordable time interval, then a more efficient way to complete the task is to go back to the beginning and start the process again. Stochastic resetting (SR)—defined as the stochastic re-initiation of a system to a reference state—represents another ubiquitous mechanism in natural search processes [9]. For instance, animal foraging often involves periodic returns to shelter or nesting sites [1], effectively “resetting” the search trajectory to balance exploration and resource exploitation [10]. Similarly, microbial cells navigating chemical gradients may periodically abandon fruitless paths via spontaneous resetting, a strategy that optimizes nutrient acquisition in heterogeneous environments [11,12]. Recent research has had recourse to SR mainly to optimize search strategies [10,13,14,15,16,17]. For example, Nagar and Gupta explored the optimal search of Brownian motion under SR with waiting times following a power-law distribution [13]; Kusmierz et al. investigated search strategy optimization for heavy-tailed Lévy flights combined with SR [14]. Such resetting events, whether driven by energy constraints, environmental cues, or internal behavioral rhythms, impose a hierarchical organization on random walks, transforming diffusive dynamics into controlled transport processes.

Recently, attention has shifted to the impact of SR on diffusion dynamics. Liu et al. investigated the diffusive behavior of Lévy walks under SR with rests [18]; Kuśmierz et al. [19] and Zhou et al. [20] studied the subdiffusive dynamics of continuous-time random walks (CTRW) under exponentially distributed stochastic resetting times, respectively. Bodrova and Sokolov explored diffusion under power-law resetting [21], but focused on CTRWs with finite step lengths, thereby neglecting the interplay between long-range jumps and power-law resetting. Targeting the long-range jump properties of Lévy walks, our study examines their behavior under power-law resetting [13,21,22], aiming to reveal novel phenomena beyond the scope of earlier CTRW models [19,20,21]. In natural systems, superdiffusive processes such as animal foraging [2] and cellular transport [4] exhibit both Lévy-type long-distance migration and power-law resetting (e.g., periodic returns to nesting sites). The approach developed in this paper provides a quantitative framework for describing such phenomena by elucidating how the exponents of the jump length and resetting-time distributions jointly govern the mean-square displacement of a tagged diffuser—thus offering a more flexible tool to interpret diffusion data in biological and physical systems.

## 2. Lévy Walks Under Stochastic Resetting

One-dimensional (1D), unbiased Lévy walks constitute a class of random walks characterized by step lengths distributed according to a Lévy distribution [8]. Numerically, symmetric Lévy distributions can be generated through the characteristic function,

(1) $$F\left(k\right)=\exp\left[-{c|k|}^{\alpha }\right],\;0<\alpha \leq 2,$$

where the scale parameter *c* may be conveniently set to unity (c≡1) and the (Lévy) index α spans the interval (0, 2]. This characteristic function corresponds to analytical expressions in the spatial domain only for three specific α values: Lévy distributions for α=1/2, Cauchy distributions for α=1, and Gaussian distributions for α=2.

Lévy-distributed random number generation necessitates numerical methods such as the Chambers–Mallows–Stuck algorithm [23] or the Mantegna algorithm [24]. The latter generates Lévy-distributed step lengths through two sets of normally distributed random numbers, providing simplified formulation and computational efficiency, though limited to symmetric distributions, such as those in Equation (1). Consequently, we had recourse to the Mantegna algorithm for all numerical simulations presented herein.

**Figure 1:**
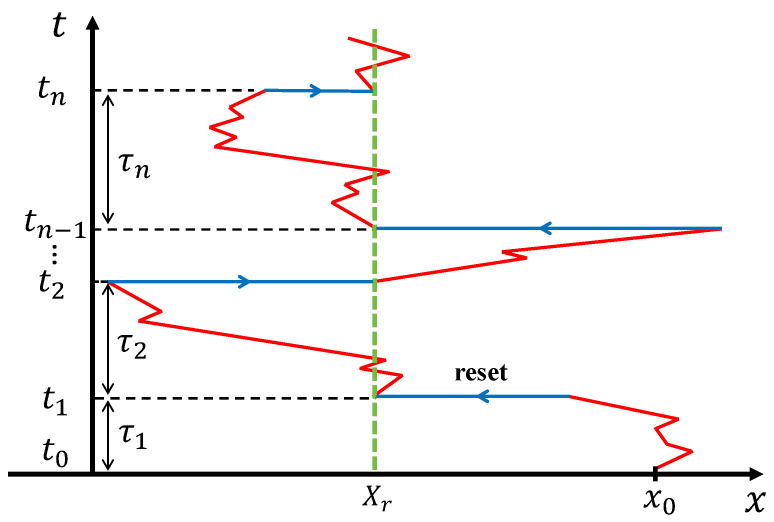
Schematic of Lévy walk under SR. $x_{0}$ denotes a random starting position, while $X_{r}$ represents the resetting position.

We conducted systematic MSD simulations for Lévy walks and compared numerical results with theoretical predictions. For each generated step length $s_{i}$, we calculated the corresponding step duration $\Delta_{i}=\left|s_{i}\right|/v$ for a particle with constant speed *v*. In our simulations, we conventionally set v≡1. Iterative application of this procedure generates 1D Lévy walk trajectories (Figure 1). Specifically, we fixed the Lévy index α and generated $N=10^{6}$ independent trajectories originating from the initial condition x(0)=0. For each trajectory, we recorded its position time series and computed the ensemble-averaged MSD

(2) $$\langle x^{2}\left(t\right)\rangle \;=\;\frac{1}{N}\sum_{k=1}^{N}{\left|x_{k}\left(t\right)-x_{k}\left(0\right)\right|}^{2},$$

with $x_{k}\left(0\right)=0$.

For an untruncated Lévy walk, the asymptotic MSD [i.e., the t→∞ limit of Equation (2)] is known to scale like [25,26]

(3) $$M_{0}\left(t\right)=\lim_{t\to \infty }\langle x^{2}\left(t\right)\rangle ∼\left\{\begin{array}{cc} \left(1-\alpha \right)\;t^{2},\; & 0<\alpha <1,\\ \frac{t^{2}}{\ln t}, & \alpha =1,\\ 2F_{\alpha }\;t^{3-\alpha }, & 1<\alpha <2,\\ 2t, & \alpha =2, \end{array}\right.$$

with $F_{\alpha }=|\Gamma \left(1-\alpha \right)|{\left(\alpha -1\right)}^{2}/\left[\alpha \;\Gamma \left(4-\alpha \right)\right]$ and Γ(…) denoting the Gamma function. We note that by setting c=v=1, we implicitly established dimensionless units for both *x* and *t*.

**Figure 2:**
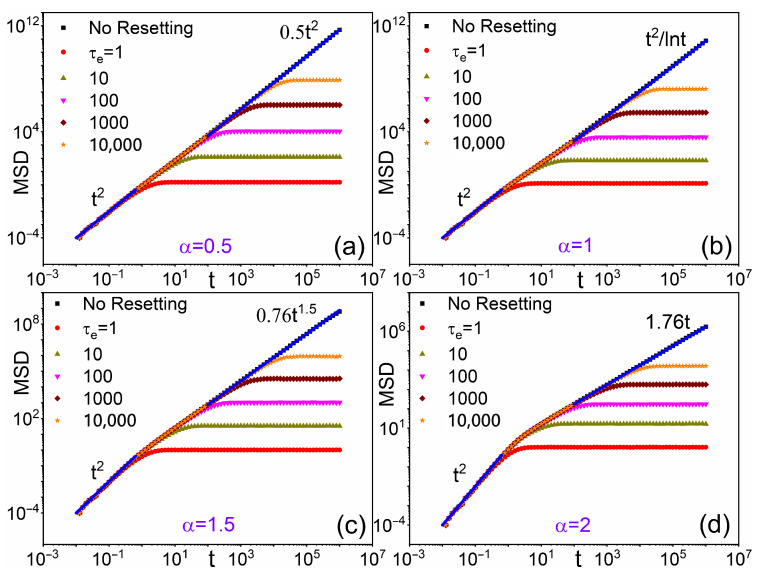
MSD of Lévy walks under exponential resetting, Equation (7), for (**a**) α=0.5; (**b**) α=1; (**c**) α=1.5; (**d**) α=2 and $\tau_{e}$ (see legends). Asymptotic fits of the free diffusion data (black symbols) are reported for a comparison with the analytical predictions of Equation (3): the *t* scaling laws appear in close agreement, while discrepancies in the prefactors are minimal. The ballistic MSD growth, MSD∼$t^{2}$ for t<1, is a numerical artifact of the Lévy step generator of Equation (1) for c=v=1—see discussion following Equation (4).

Except for α=1, the 1D asymptotic Lévy walk diffusion can be formulated as follows:

(4) $$M_{0}\left(t\right)=Kt^{\gamma_{0}},$$

where the free diffusion prefactor *K* and exponent $\gamma_{0}$ are functions of α that can be directly obtained from Equation (3). However, in view of Equation (1), at short times, ct<1 (i.e., t<1), the particle propagates at constant speed, v=1, yielding $\langle x^{2}\left(t\right)\rangle ≃t^{2}$ independently of α, as demonstrated by the short-time dat fits in Figure 2. This effect is an artifact of our Lévy step generator and is not further considered in the following.

We employed Equation (4) to fit simulated MSD curves and extract numerical estimates for *K* and $\gamma_{0}$, as summarized in Table 1. Notwithstanding the increased systematic errors in the Mantegna algorithm for 1<α<2, all relative errors remained below 20%, confirming numerical reliability. In particular, simulated exponents consistently matched theoretical predictions across repeated trials, validating our numerical code for subsequent Lévy walk simulations under SR conditions. We confirm in passing that, as expected [27], for Lévy walks with an intermediate index range, 1<α<2, the non-Gaussian character of the *x*-coordinate distribution gives rise to two distinct notions of squared length scale: the MSD and a characteristic squared length. Both quantities grow unboundedly in time as power laws, but with exponents so close that they are difficult to resolve with current statistical precision.

Under SR, the Lévy walker is reset to the initial position $X_{r}$ at random time intervals τ (as illustrated in Figure 1) drawn from a distribution function ψ(τ), to be specified. In 1D, its coordinate can be expressed as follows:

(5) $$\begin{array}{c} x\left(t\right)=X_{r}+\tilde{x}\left(t-t_{n-1}\right)\;\;\;\mathrm{for}\;\;\;t\in \left[t_{n-1},t_{n}\right), \end{array}$$

where $t_{n-1}$ and $t_{n}$ denote the times of two consecutive random resetting events and $\tilde{x}\left(t\right)$ represents the same Lévy walk reinitialized with $\tilde{x}\left(0\right)=0$ (Method I in notation of Ref. [19]) and truncated after the resetting interval $\tau_{n}=t_{n}-t_{n-1}$. In our simulations, we set $X_{r}=0$ and, contrary to the authors of Ref. [28], we allowed resetting to occur at any time, i.e., also during Lévy steps (continuous SR).

As an Ansatz for our analysis, we postulate that the asymptotic MSD under resetting obeys the same scaling law, Equation (4), as for a free Lévy walker, as follows:

(6) $$M\left(t\right)=\lim_{t\to \infty }\langle x^{2}\left(t\right)\rangle =K^{\prime }t^{\gamma },$$

with appropriate prefactor $K^{\prime }$ and diffusion exponent γ to be determined based on the specific choice of ψ(τ). Logarithmic corrections to the aforementioned scaling law, occurring for α=1 according to Equation (3), and for certain power laws of the τ distributions, will be treated separately.

Finally, we note that for anomalous (ultraslow) diffusive processes under SR, the time-averaged MSD may differ from the ensemble-averaged MSD [29]. The discussion of such non-ergodic regimes falls outside the scope of the present work. Nevertheless, even in those cases ergodicity may be recovered by suitably tuning the reset rate or protocol—particularly when the waiting-time distribution of resets possesses finite moments. To this end, tools such as the instantaneous MSD have been introduced [30], offering a crucial diagnostic for single-particle tracking experiments in biophysics.

## 3. Lévy Diffusion Under Exponential Stochastic Resetting

In this section, we revisit Lévy walks subject to SR with exponentially distributed resetting times [18,19,20]. Our goal is twofold: first, to develop and validate the renewal theory framework, which will be extended in Section 4 to the more complex case of power-law resetting; and second, to use exponential resetting as a benchmark to illustrate how the interplay between the power-law statistics of Lévy walk steps and resetting times gives rise to distinct diffusive regimes.

We begin by assuming that the resetting times, τ, follow an exponential distribution,

(7) $$\psi \left(\tau \right)=\frac{1}{\tau_{e}}e^{-\tau /\tau_{e}},$$

with finite first and second moments, respectively, as follows:

(8) $$\langle \tau \rangle =\tau_{e}\;\;\;\;\mathrm{and}\;\;\;\;\langle \tau^{2}\rangle =2\tau_{e}^{2}.$$

Figure 2 illustrates how exponential resetting influences the temporal evolution of Lévy walk MSD for various α values. Colored symbols represent MSD data for different resetting parameters $\tau_{e}$, while black symbols denote the corresponding MSD in the absence of resetting. For $t\ll \tau_{e}$, low resetting probability results in MSD closely approximating free diffusion. More significantly, for $t∼\tau_{e}$, frequent resetting induces MSD curve deviations from free growth, ultimately leading to horizontal saturation plateaus for $t\gg \tau_{e}$. The plateau heights depend on both the Lévy index α and resetting parameter $\tau_{e}$. This phenomenon demonstrates that regardless of underlying dynamics (ballistic, superdiffusive, etc.), exponential stochastic resetting establishes an upper bound for MSD at long times, preventing unbounded diffusion [10,16].

The exact expressions for the dependence of Lévy walk MSD on the tunable parameters α and $\tau_{e}$ are derived using a renewal theory method and numerically validated in the following subsection.

### Scaling Analysis and Diffusion Transition Times

Most SR protocols assume that the contribution of each resetting event to the tracer’s MSD can be treated independently. More elaborate protocols, such as the one analyzed in Ref. [19], go beyond this simplification. In the present work, we model the resetting process defined in Equation (5) as a renewal process [31], for which the dynamics restarts statistically afresh after each reset, ensuring temporal homogeneity between resetting events. The renewal theory provides a concise and straightforward analytical framework. The general renewal equation for the MSD in the asymptotic regime follows:

(9) $$M\left(t\right)=S\left(t\right)M_{0}\left(t\right)+\int_{0}^{t}\psi \left(\tau \right)\;M\left(t-\tau \right)\;d\tau ,$$

where $M_{0}\left(t\right)$ is the known free MSD of Equation (3) and ψ(τ) is an arbitrary resetting time distribution function. S(t) is the survival function associated with ψ(τ), i.e., the probability that resetting happens after a waiting time larger than *t*,

(10) $$S\left(t\right)=\int_{t}^{\infty }\psi \left(\tau \right)\;d\tau .$$

When the mean 〈τ〉 of the resetting time distribution ψ(τ) exists (i.e., is finite), one can make use of the closed form of the asymptotic “age distribution”,

(11) $$f_{\mathrm{age}}\left(t\right)=\frac{S(t)}{\langle \tau \rangle },$$

to formally solve Equation (9) for t≫〈τ〉 [32], as follows:

(12) $$M\left(t\right)=\int_{0}^{t}f_{\mathrm{age}}\left(t^{\prime }\right)M_{0}\left(t^{\prime }\right)dt^{\prime }.$$

In this way, the asymptotic MSD is formulated in terms of the average Lévy diffusion within single resetting events.

Equation (12) provides the most direct way to calculate the plateau $M_{\mathrm{st}}\equiv \lim_{t\to \infty }M\left(t\right)$ for the asymptotic Lévy MSD under exponential resetting. From the exponential distribution in Equation (7), we obtain the survival function $S\left(t\right)=e^{-t/\tau_{e}}$ and the corresponding age distribution $f_{\mathrm{age}}\left(t\right)=e^{-t/\tau_{e}}/\tau_{e}$. Accordingly, for α≠1, Equation (12) can be rewritten as follows:

(13) $$M_{\mathrm{st}}\equiv \lim_{t\to \infty }M\left(t\right)=\frac{K}{\tau_{e}}\int_{0}^{\infty }t^{\gamma_{0}}e^{-t/\tau_{e}}\;dt,$$

where we used the formal expression for $M_{0}\left(t\right)$ introduced in Equation (4). This integral can be easily performed in terms of Gamma functions, to obtain the following:

(14) $$M_{\mathrm{st}}=K\;\tau_{e}^{\gamma_{0}}\;\Gamma \left(1+\gamma_{0}\right),\;\alpha \neq 1.$$

For α=1, the Lévy walk MSD in Equation (3) does not follow a power law, so we must handle it separately. Inserting the free MSD expression, $M_{0}\left(t\right)=t^{2}/\ln t$ for α=1, into Equation (12), we end up with an integral expression for $M_{\mathrm{st}}$:

(15) $$M_{\mathrm{st}}=\int_{0}^{\infty }\frac{1}{\tau_{e}}e^{-t/\tau_{e}}\;\frac{t^{2}}{\ln t}\;dt.$$

[We recall that *x* and *t* are expressed in the dimensionless units corresponding to c=v=1]. For $\tau_{e}\gg 1$, this integral can be separated into two distinct integrals by introducing the auxiliary variable $y=t/\tau_{e}$, as follows:

(16) $$M_{\mathrm{st}}=\frac{\tau_{e}^{2}}{\ln\tau_{e}}\int_{0}^{\infty }\left(1-\frac{\ln y}{\ln\tau_{e}}\right)y^{2}e^{-y}\;dy,$$

where $\int_{0}^{\infty }y^{2}e^{-y}\;dy=2$ and $\int_{0}^{\infty }y^{2}\ln y\;e^{-y}\;dy=2\psi \left(3\right)$, with $\psi \left(z\right)=\frac{d}{dz}\ln\Gamma \left(z\right)$. For an integer *n*, $\psi \left(n\right)=-\gamma_{E}+\sum_{z=1}^{n-1}1/z,$ where $\gamma_{E}\approx 0.5772$ is the Euler–Mascheroni constant, then, for n=3, ψ(3)≈1. In conclusion, Equation (16), can be approximated as follows:

(17) $$\begin{array}{cc} M_{\mathrm{st}} & ≃\frac{2\tau_{e}^{2}}{\ln\tau_{e}}\left(1-\frac{1}{\ln\tau_{e}}\right),\;\alpha =1. \end{array}$$

Deriving the superdiffusive regime of M(t) is generally a more complex task, requiring the solution of the full renewal Equation (9). In the case of exponential SR, we need to extract the time dependence of M(t) for $t\ll \tau_{e}$. Upon Laplace transformation, Equation (9) can be equivalently reformulated as follows:

(18) $$\tilde{M}\left(s\right)={\tilde{S}}_{0}\left(s\right)+\tilde{\psi }\left(s\right)\;\tilde{M}\left(s\right)\;⟹\;\tilde{M}\left(s\right)=\frac{{\tilde{S}}_{0}\left(s\right)}{1-\tilde{\psi }\left(s\right)},$$

where ${\tilde{S}}_{0}\left(s\right)=L\left\{S\left(t\right)M_{0}\left(t\right)\right\}\equiv L\left\{S_{0}\left(t\right)\right\}$. The large-*t* (short-*t*) behavior of M(t) can be readily determined by analyzing Equation (18) in the limit of small (large) *s*.

Using Equation (4) for $M_{0}\left(t\right)$ with α≠1 and the explicit expression for S(t) reported above, calculating $\tilde{M}\left(s\right)$ is reduced to evaluating the Laplace transform of elementary functions (powers and exponentials); hence, $\tilde{M}\left(s\right)=K\;\Gamma \left(1+\gamma_{0}\right)\tau_{e}^{\gamma_{0}}/\left[s{\left(1+s\tau_{e}\right)}^{\gamma_{0}}\right]$. Inverse transforming $\tilde{M}\left(s\right)$ yields the following:

(19) $$M\left(t\right)=M_{0}\left(t\right),\;t\ll \tau_{e},$$

as anticipated in Figure 2. Of course, in the opposite limit, $t\gg \tau_{e}$, one recovers the plateau value of Equation (14). The same conclusion is readily extended to the case of logarithmic corrections to the scaling Equation (4) for $M_{0}\left(t\right)$, which corresponds to the marginal case α=1 in Equation (3).

**Figure 3:**
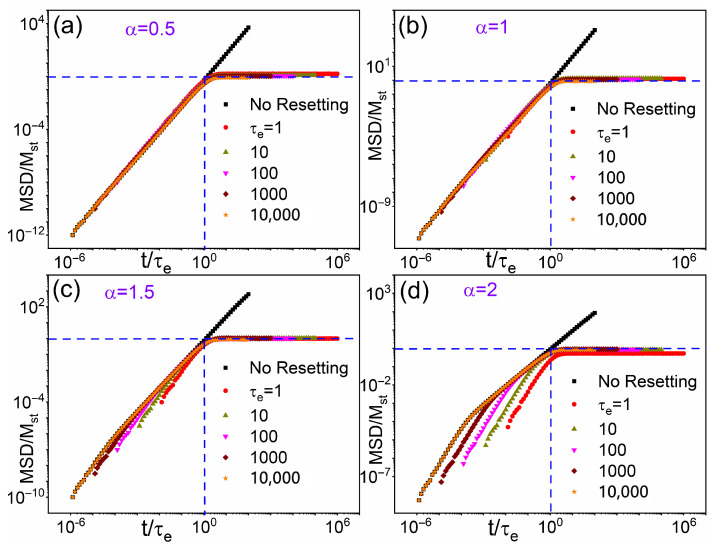
Rescaled data from Figure 2: $\mathrm{MSD}/M_{\mathrm{st}}$, vs. rescaled time, $t/\tau_{e}$, for (**a**) α=0.5; (**b**) α=1; (**c**) α=1.5; and (**d**) α=2. $M_{\mathrm{st}}$ was computed from Equations (14) for α≠1 and (17) for α=1, by inserting the numerical values of *K* obtained by fitting the simulation data for free Lévy walks; see Table 1.

To validate our analytical predictions, in Figure 3 we display the datasets from Figure 2 with *t* and MSD rescaled respectively by $\tau_{e}$ and $M_{\mathrm{st}}$. For $M_{\mathrm{st}}$, we utilized the appropriate analytical expressions from Equations (14) and (17), employing numerical values for *K* obtained from simulation data fitting [rather than theoretical values from Equation (3)]. This approach ensures greater consistency, accounting for numerical inaccuracies inherent in Mantegna’s method for Lévy step generation (Table 1). For comparative purposes, we include in each panel (i.e., for all α values) the corresponding superdiffusive curve (black) in the absence of SR. For graphical convenience, *t* and the no-resetting MSD data have been rescaled by $\tau_{e}=10^{4}$ and the corresponding $M_{\mathrm{st}}$, respectively. All colored curves for finite $\tau_{e}$ converge asymptotically to $\mathrm{MSD}/M_{\mathrm{st}}\approx 1$ for $t\gg \tau_{e}$, in close agreement with theory. This occurs because, when the observation time exceeds the mean SR time, $\tau_{e}$, accumulated reset events drive the system from free superdiffusion into a regime of reset-governed localization. In contrast, Lévy diffusion remains largely insensitive to SR for $t<\tau_{e}$. This establishes a robust reference framework for subsequent comparisons under varying reset distributions.

**Figure 4:**
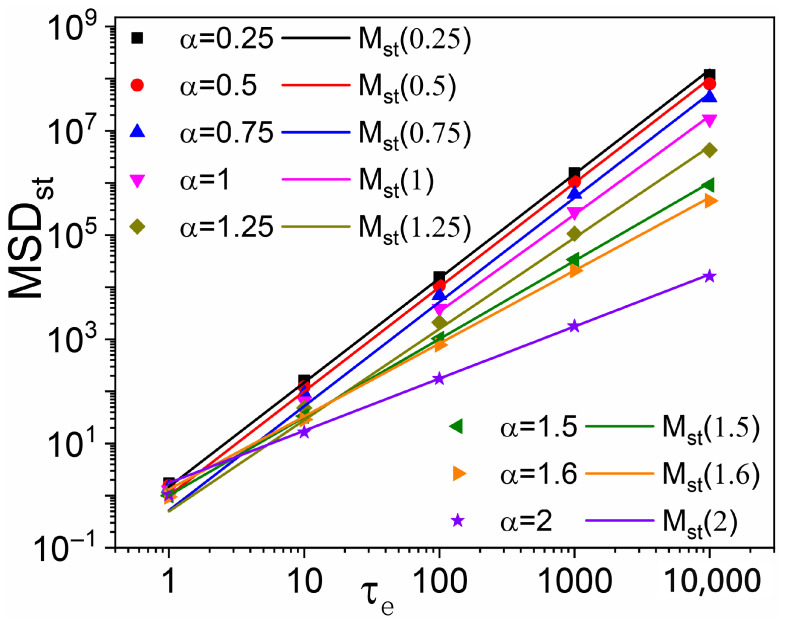
Lévy walk ${\mathrm{MSD}}_{\mathrm{st}}$ vs. $\tau_{e}$ under exponential SR for different α: numerical simulation data (symbols) vs. the relevant theoretical predictions of Section 3 (straight lines). Simulation data and analytical predictions appear to clearly disagree for $\tau_{e}≲10$.

Finally, Figure 4 illustrates, for various Lévy indices α, the dependence of $M_{\mathrm{st}}$ on the mean resetting time $\tau_{e}$. Colored points represent simulation-measured asymptotic ${\mathrm{MSD}}_{\mathrm{st}}$; solid lines of the same color denote the corresponding theoretical predictions for $M_{\mathrm{st}}$ from Equations (14) and (17). Our Lévy walk simulations result in the close overlap of the theoretical curves for $M_{0}\left(t\right)$ in Equation (3), though only for t≳100 (see Figure 2). Therefore, not surprisingly, excellent agreement between numerical data and analytical predictions is achieved in Figure 4 for $\tau_{e}≳100$ across the entire α range.

## 4. Lévy Diffusion Under Power-Law Stochastic Resetting

The exponential distribution of resetting times, given in Equation (7), is commonly adopted in the literature to model scenarios where a searcher must intermittently return to a base location, for example, to rest or refuel. Such behavior naturally arises when interruptions (e.g., due to accidents or resource depletion) follow a Poisson process. However, in many complex systems, resetting intervals are better described by heavy-tailed distributions. For instance, pause durations in animal foraging often exhibit power-law or multi-scale statistics [33], and inter-spike intervals in neuronal networks can also display similar multi-scale temporal dynamics [34]. These observations motivate our next step: a detailed analysis of stochastic resetting with power-law-distributed resetting times.

Let the Lévy walker be now instantaneously reset to $X_{r}=0$ after a random time τ drawn from a Pareto (Type I) distribution, as follows:

(20) $$\psi \left(\tau \right)=\left\{\begin{array}{cc} \frac{\beta }{\tau_{0}}{\left(\frac{\tau_{0}}{\tau }\right)}^{1+\beta },\; & \tau \geq \tau_{0},\\ 0, & \tau <\tau_{0}, \end{array}\right.$$

where $\tau_{0}$ denotes a scale parameter and exponent β characterizes the distribution tail decay. The τ first and second moments,

(21) $$\begin{array}{cc} \langle \tau \rangle & =\frac{\beta \tau_{0}}{\beta -1},\;\beta >1\\ \langle \tau^{2}\rangle & =\frac{\beta \tau_{0}^{2}}{\beta -2},\;\beta >2, \end{array}$$

diverge for β<1 and β<2, respectively. In our simulations, unless otherwise specified, we assume a small resetting timescale, namely $\tau_{0}=0.1$. The principal findings of this section can be readily generalized to Lomax distributions with identical $\tau_{0}$ and β parameters, and extended in principle to other heavy-tailed τ distributions.

**Figure 5:**
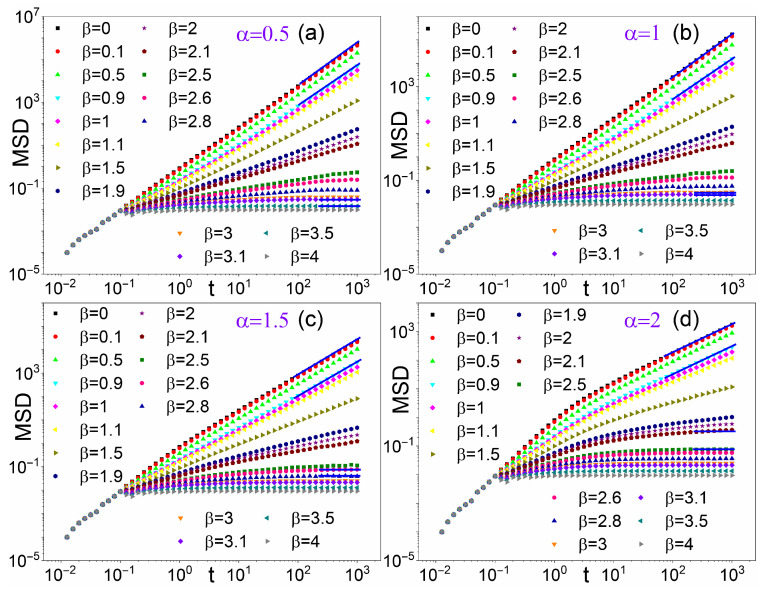
MSD vs. time *t* for Lévy walks under power-law SR for (**a**) α=0.5; (**b**) α=1; (**c**) α=1.5; and (**d**) α=2. Different colors/symbols denote different β exponents of the Pareto distribution with $\tau_{0}=0.1$ (β=0 corresponds to no resetting).

Figure 5 illustrates the temporal evolution of Lévy walk MSD under Pareto-distributed resetting times for various Lévy indices α. Colored curves for different β values are plotted in each panel; black curves for β=0 represent free Lévy diffusion. In each panel, MSD curves for β<1 run parallel to a black curve for β=0, exhibiting approximate free Lévy superdiffusive scaling. As β increases, the diffusion exponent gradually decreases until, above a critical β threshold, MSD reaches an asymptotic plateau, indicating complete diffusion suppression. This critical β threshold appears to decrease with α. All of the curves of all the panels coincide at short times as for $t<\tau_{0}=0.1$, resetting has not yet commenced, while, for t<1, the walk is purely ballistic and α insensitive. At long times, the curves in each panel diverge, demonstrating specific SR diffusion behavior.

Figure 5 graphically demonstrates the strong correlation between exponents α and β, which will be analyzed in detail in the forthcoming subsections. As anticipated in Section 2, we aim to determine how SR modifies the prefactor and exponent of the free MSD, $M_{0}\left(t\right)$ from Equation (4). For this purpose, we must exclude from our analysis Lévy walks with α=1, where Equation (3) indicates that additional logarithmic time dependence must be incorporated. These cases will be addressed separately in Section 4.4.

### 4.1. 0<β≤1: SR Independent Diffusion Exponent

As the mean resetting time in Equation (21) diverges for β<1, we start our analysis from the Laplace transform of the full renewal equation in Equation (18). Here, the survival function of the Pareto distribution of Equation (20), reads

(22) $$S\left(t\right)=\left\{\begin{array}{cc} \int_{t}^{\infty }\psi \left(\tau \right)d\tau ={\left(\frac{\tau_{0}}{t}\right)}^{\beta },\; & t\geq \tau_{0},\\ 1, & t<\tau_{0}. \end{array}\right.$$

Using Equation (4) for $M_{0}\left(t\right)$ and neglecting contributions to S(t) for $t<\tau_{0}$, we can approximate $S_{0}\left(t\right)=S\left(t\right)M_{0}\left(t\right)$ to $S_{0}\left(t\right)=K\tau_{0}^{\beta }t^{\gamma_{0}-\beta }$ for α≠1 and calculate its Laplace transform,

(23) $${\tilde{S}}_{0}\left(s\right)=\tau_{0}^{\beta }\Gamma \left(1+\gamma_{0}-\beta \right)s^{\beta -(1+\gamma_{0})}.$$

**Figure 6:**
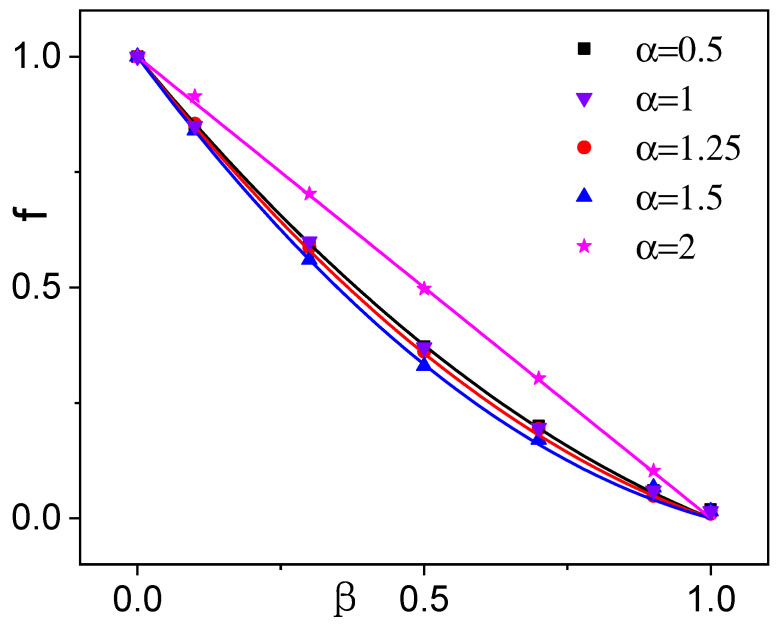
Relative change of the MSD prefactor, $f=K^{\prime }/K$, vs. β in the range 0<β<1 for different α. Symbols: simulation results; solid lines of the same color: analytical predictions from Equation (27). Resetting timescale, $\tau_{0}=0.1$, as in Figure 5.

**Figure 7:**
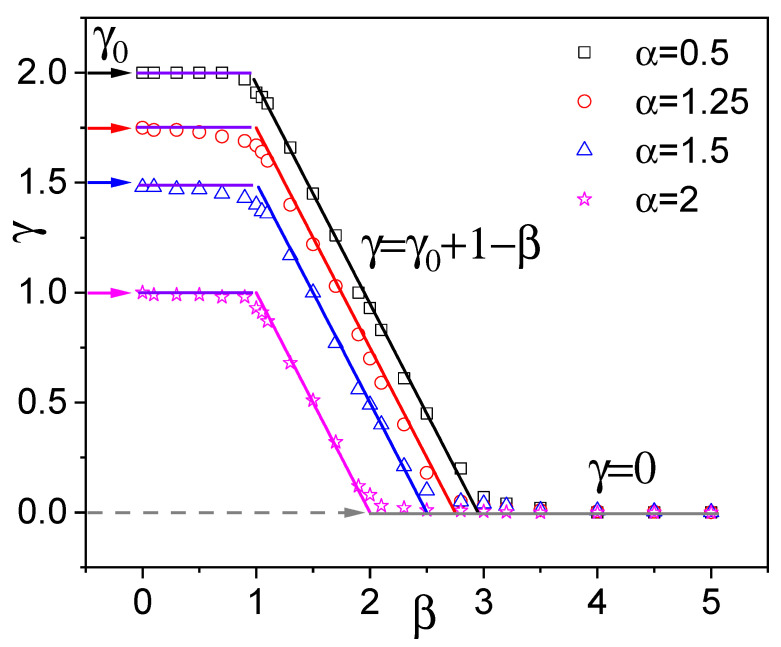
Diffusion exponent γ vs. β for different Lévy indices α. Symbols: simulation results; solid lines of the same color: theoretical prediction of Equation (33) (color code in the legend). Pareto resetting timescale $\tau_{0}=0.1$.

**Figure 8:**
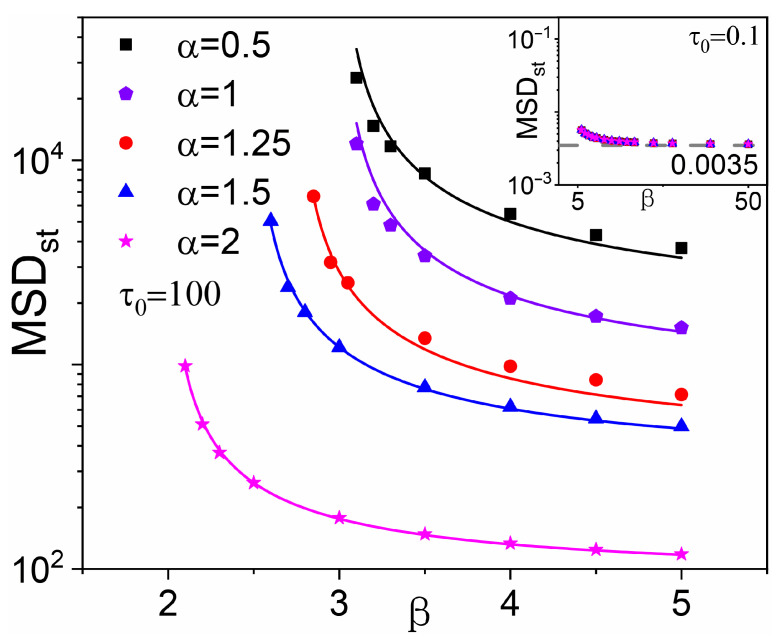
MSD plateau vs. β for $\beta >\gamma_{0}+1$, $\tau_{0}=100$, and different α. Symbols represent the numerical results for MSD_st_; solid lines represent the corresponding theoretical predictions, $M_{\mathrm{st}}$ from Equation (36). Inset: MSD_st_ for large β at $\tau_{0}=0.1$.

Before proceeding, we note that this analysis assumes that the free asymptotic MSD given in Equation (4) remains valid for all $t>\tau_{0}$. However, when comparing with numerical simulations, this assumption is physically justified only when $\tau_{0}>c/v$, where c/v=1 is the intrinsic timescale of the Lévy walk defined in Equation (1). The MSD data presented here for Pareto-distributed resetting times were obtained with $\tau_{0}=0.1$, i.e., well below the intrinsic scale. As apparent in Figure 5d, a clear power-law growth of M(t) emerges only for times *t* exceeding both $\tau_{0}$ and c/v, confirming that the asymptotic regime is governed by the larger of the two timescales. Nevertheless, as shown in Figure 6, Figure 7 and Figure 8, the numerical estimates of the prefactors in M(t) are closely reproduced by the above assumption, thereby underscoring the role of $\tau_{0}$ as a cutoff timescale.

The Laplace transform of the Pareto distribution is special at β=1 (critical point) and needs to be calculated separately. Let us calculate first the Laplace transform of the Pareto distribution for 0<β<1 and s→0:

(24) $$\tilde{\psi }\left(s\right)=1-\Gamma \left(1-\beta \right)\tau_{0}^{\beta }s^{\beta }+o\left(s^{\beta }\right).$$

Substituting ${\tilde{S}}_{0}\left(s\right)$, Equation (23), and $\tilde{\psi }\left(s\right)$, Equation (24), into Equation (18) yields the following:

(25) $$\tilde{M}\left(s\right)=K\;\frac{\Gamma (1+\gamma_{0}-\beta )}{\Gamma (1-\beta )}\;s^{-(1+\gamma_{0})}.$$

Finally, taking the inverse Laplace transform of $\tilde{M}\left(s\right)$ in Equation (25), we obtain an analytical expression for the asymptotic Lévy walk MSD under power-law resetting of the form anticipated in Equation (6), with

(26) $$\gamma =\gamma_{0},\;\;0<\beta <1,$$

and

(27) $$K^{\prime }=K\frac{\Gamma (1+\gamma_{0}-\beta )}{\Gamma \left(1-\beta \right)\;\Gamma \left(1+\gamma_{0}\right)}.$$

We immediately observe that, while the diffusion exponent for 0<β<1 remains invariant, the prefactor $K^{\prime }$ strongly depends on β. The invariance of the diffusion exponent γ arises because the mean resetting time 〈τ〉 diverges; consequently, the asymptotic form of the MSD in Equation (3) is reached before SR significantly influences the dynamics.

Next, we consider the special case of β=1. For s→0, the Laplace transform of the Pareto distribution with β=1 reads

(28) $$\tilde{\psi }\left(s\right)=1-s\tau_{0}\left[\ln\left(\frac{1}{s\tau_{0}}\right)+\gamma_{E}-1\right]+O\left(s^{2}\ln s\right).$$

Inserting Equations (23) and (28) into Equation (18), we obtain the following:

(29) $$\tilde{M}\left(s\right)∼K\Gamma \left(\gamma_{0}\right)\frac{s^{-(\gamma_{0}+1)}}{\ln\left(1/s\right)},$$

whence, upon inverse Laplace transformation,

(30) $$M\left(t\right)∼K\frac{\gamma_{0}t^{\gamma_{0}}}{\ln t},\;\beta =1.$$

In Figure 6 we compare our analytical predictions for the relative prefactor change, $f=K^{\prime }/K$, with extensive numerical simulation results. The overall agreement is satisfactorily close. We observe that as β→0, resetting events become increasingly rare, and *f* approaches unity, indicating that SR becomes ineffective. Conversely, as β increases from zero to one, *f* decreases monotonically to zero, suggesting the suppression of unbounded diffusion. The β dependence of *f* varies with the index α; in particular, for α=2 (Brownian walk), *f* decreases linearly with β, as first reported in Ref. [35].

### 4.2. $1<\beta <\gamma_{0}+1$: Diffusion Exponent Attenuation

When β>1, the mean value 〈τ〉, Equation (21), of the Pareto distribution is finite. This means that, as discussed in Section 3, the renewal Equation (9) can be solved in terms of the age distribution to obtain the approximate solution of Equation (12). The age distribution for the Pareto distribution can be readily obtained by inserting Equations (21) and (22) into Equation (11), as follows:

(31) $$f_{\mathrm{age}}\left(t\right)=\left\{\begin{array}{cc} \frac{1}{\langle \tau \rangle }{\left(\frac{\tau_{0}}{t}\right)}^{\beta },\; & \tau \geq \tau_{0},\\ \frac{1}{\langle \tau \rangle }, & \tau <\tau_{0}, \end{array}\right.$$

Upon substituting $M_{0}\left(t\right)$ from Equation (4) and $f_{\mathrm{age}}\left(t\right)$ from Equation (31) into Equation (12) for α≠1, M(t) can be written as follows:

(32) $$M\left(t\right)=\frac{K}{\langle \tau \rangle }\int_{0}^{\tau_{0}}y^{\gamma_{0}}dy+\frac{K\tau_{0}^{\beta }}{\langle \tau \rangle }\int_{\tau_{0}}^{t}y^{\gamma_{0}-\beta }dy.$$

In the limit t→∞, this integral expression must be treated differently based on the value of β. When $1<\beta <\gamma_{0}+1$, the second integral does not converge. Neglecting the first integral, the asymptotic expression for M(t) can thus be formulated again according to Equation (6), with

(33) $$\gamma =\gamma_{0}+1-\beta ,\;1<\beta <\gamma_{0}+1,$$

and

(34) $$K^{\prime }=K\tau_{0}^{\beta -1}\frac{\beta -1}{\beta (\gamma_{0}-\beta +1)}.$$

The dependence of the diffusion exponent in Equation (33) on the parameters α and β reveals that the dominance of rare, long flights, characterized by the exponent $\gamma_{0}$, is progressively suppressed as the rate of Pareto-distributed resetting events increases.

In contrast, for $\beta =\gamma_{0}+1$, the second integral in Equation (32) diverges logarithmically, so that

(35) $$M\left(t\right)∼K\frac{\beta -1}{\beta }\tau_{0}^{\beta -1}\ln t,\;\beta =\gamma_{0}+1.$$

Unlike Section 4.1, in this β regime, SR attenuates the diffusion exponent from $\gamma_{0}$ to $\gamma =\gamma_{0}+1-\beta$. In Equation (6), the limit γ→0+ when $\beta \to \gamma_{0}+1$ from below, disguises a logarithmic divergence of M(t). As demonstrated in the following subsection, when the same limit of β is approached from above, γ→0 indeed implies localization.

Figure 7 illustrates the dependence of diffusion exponent γ on Pareto exponent β for various α values. For β<1, all curves are horizontal, indicating that the diffusion exponent remains constant. When $1<\beta <\gamma_{0}+1$, the diffusion exponent decreases linearly, as predicted by Equation (33). The transition from superdiffusion to localization is clearly evident as β increases beyond $\gamma_{0}+1$, with MSD from numerical simulations saturating to a constant plateau for $\beta >\gamma_{0}+1$.

### 4.3. $\beta >\gamma_{0}+1$: Localization

When $\beta >\gamma_{0}+1$, both integrals in Equation (32) converge as t→∞, so that the Lévy MSD under SR now approaches a plateau. The direct integration of Equation (32) yields the following:

(36) $$M_{\mathrm{st}}=K\tau_{0}^{\gamma_{0}}\frac{\beta -1}{\left(\gamma_{0}+1\right)\left(\beta -\gamma_{0}-1\right)},\;\beta >\gamma_{0}+1.$$

Figure 8 compares diffusion plateaus MSD_st_ in the localization regime, $\beta >\gamma_{0}+1$, with the analytical estimate $M_{\mathrm{st}}$ from Equation (36). The agreement is excellent across the entire α range. It can be observed that the plateau appears to diverge as $\beta \to \gamma_{0}+1$ from above, which is consistent with the logarithmic divergence of M(t), Equation (35), when the same limit is approached from below. The inset shows $M_{\mathrm{st}}$ for large β and $\tau_{0}=0.1$. As β→∞, $\langle \tau \rangle \to \tau_{0}$ [Equation (21)], which implies a high resetting frequency, with τ close to $\tau_{0}$. Consequently, the plateau onset time is also of order $\tau_{0}$. With c=v=1 in the Lévy step generator [Equation (1)], frequent resetting events with τ<1 confine the walker to the short-time ballistic-diffusive regime $M\left(t\right)=t^{2}$ (independent of α) described in Section 2. This technical limitation can be circumvented by setting $\tau_{0}\gg 1$; in Figure 8, consistently with the discussion of Figure 4, we set $\tau_{0}=100$. However, it is possible to validate Equation (36) even for $\tau_{0}<1$. Indeed, adopting ballistic parameter values, K=1 and $\gamma_{0}=2$, in Equation (4), Equation (36) predicts M_st_ = 0.0033, in close agreement with the simulation result 0.0035.

### 4.4. The Case α=1

In the marginal case with α=1, $M_{0}\left(t\right)$ exhibits logarithmic divergence, as shown in Equation (3). This implies that asymptotic power-law scaling is untenable for the Lévy walk MSD both in the absence [Equation (4)] and presence [Equation (6)] of SR. For this reason, Lévy walks with α=1 must be treated separately. Simulation data for 0<β<1 in Figure 6 suggest the convergence of the *t* function $M\left(t\right)/M_{0}\left(t\right)$ toward a finite ratio, *f*, also in this case.

To explain this result, we repeated the analytical procedure of Equations (23)–(27) for $M_{0}\left(t\right)=t^{2}/\ln t$ instead of $M_{0}\left(t\right)=Kt^{\gamma_{0}}$. In the limit of small *s*, one obtains

(37) $$\tilde{M}\left(s\right)=\frac{{\tilde{S}}_{0}\left(s\right)}{1-\tilde{\psi }\left(s\right)}\approx \frac{\Gamma (3-\beta )}{\Gamma (1-\beta )}\cdot \frac{s^{-3}}{-\ln s},$$

whose inverse Laplace transform,

(38) $$M\left(t\right)∼\frac{\Gamma (3-\beta )}{2\Gamma (1-\beta )}\frac{t^{2}}{\ln t},\;\alpha =1,\;0<\beta <1,$$

scales with time exactly like $M_{0}\left(t\right)$. The relative change in the prefactor, f=Γ(3−β)/[2Γ(1−β)] coincides with that obtained from Equation (27) for 0<α<1, i.e., for $\gamma_{0}=2$, as shown in Figure 6.

Stochastic resetting diffusion attenuation for α=1 is expected to occur in the Pareto exponent range 1<β<3—we remind the reader here that $\gamma_{0}\to 2$ as α→1 from both below and above. Adopting the procedure of Section 4.2 for $M_{0}\left(t\right)=t^{2}/\ln t$ leads to replacing Equation (32) with

(39) $$M_{\mathrm{st}}=\frac{1}{\langle \tau \rangle }\int_{0}^{\tau_{0}}\frac{y^{2}}{\ln y}dy+\frac{\tau_{0}^{\beta }}{\langle \tau \rangle }\int_{\tau_{0}}^{t}\frac{y^{2-\beta }}{\ln y}dy.$$

On following the integration procedure adopted in Section 3, one recognizes immediately that the asymptotic regime of M(t) is governed by the diverging second integral, as follows:

(40) $$M\left(t\right)\approx \tau_{0}^{\beta -1}\frac{\beta -1}{\beta (3-\beta )}\frac{t^{3-\beta }}{\ln t},\;\alpha =1,\;1<\beta <3.$$

Comparing this result with the input-free MSD, $M_{0}\left(t\right)=t^{2}/\ln t$, indicates that the *t*-exponent and the prefactor in Equation (40) coincide with γ and $K^{\prime }$, respectively, from Equations (33) and (34), for K=1 and $\gamma_{0}=2$.

Finally we consider SR localization in the range β>3. An estimate for the MSD plateaus, MSD_st_, is also analytically tractable. One simply needs to take the limit t→∞ of Equation (39) to obtain the following:

(41) $$M_{\mathrm{st}}≃\frac{\tau_{0}^{2}\left(\beta -1\right)}{3\left(\beta -3\right)\;|\ln\tau_{0}|},\;\alpha =1,\;\beta >3.$$

This estimate for $M_{\mathrm{st}}$ is also in good agreement with the simulation, as demonstrated in Figure 8.

The special cases β=1 [Equation (38)] and β=3 [Equation (40)] for α=1 required us to firstly solve Equations (18) and (12), respectively, for the specific memory kernel $M_{0}\left(t\right)=t^{2}/\ln t$. Through laborious analytical calculations, we obtained approximate expressions for M(t) in the large-*t* regime, as follows:

(42) $$M\left(t\right)≃\frac{1}{2}\;\frac{t^{2}}{{\left(\ln t\right)}^{2}},\;\alpha =1,\;\beta =1,$$

and

(43) $$M\left(t\right)≃\frac{2\tau_{0}^{2}}{3}\;\ln\left(\ln t\right),\;\alpha =1,\;\beta =3,$$

which are consistent with the data from numerical simulations displayed in Figure 5b. In conclusion, as in the case of α≠1, SR tends to weaken the superdiffusive behavior of the Lévy walk for β≥1, while for 0<β<1 it only modifies the prefactor.

## 5. Conclusions

In this paper we investigated the diffusive dynamics of Lévy walks under stochastic resetting with Pareto distributions of the resetting times. Analytical expressions for the asymptotic mean squared displacement (MSD) of Lévy walks under power-law resetting are derived using renewal theory, and validated through numerical simulations.

**Figure 9:**
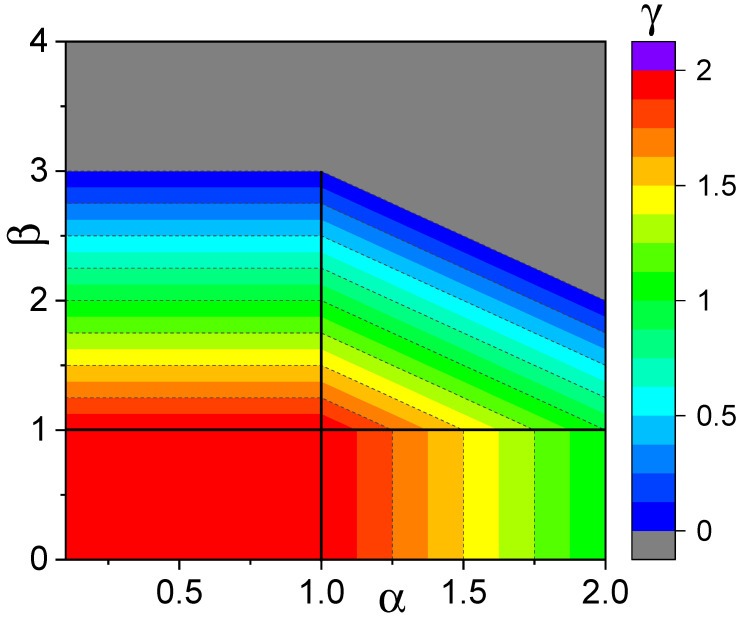
Diffusion exponent γ vs. Lévy index α and stochastic resetting exponent β (see color bar). The gray region corresponds to resetting induced localization.

Our main results are summarized in the 2D phase diagram in Figure 9. There, we display the dependence of the diffusion exponent γ as a function of the Lévy index α, and resetting-time distribution exponent β. The horizontal axis α, ranging from 0 to 2, is divided into two segments: for 0<α<1, the MSD of the free Lévy walk (β=0) exhibits ballistic growth, i.e., $\langle x^{2}\rangle ∼t^{2}$; for 1<α<2, the free MSD behaves as $\langle x^{2}\rangle ∼t^{3-\alpha }$; and, finally, α=2 corresponds to the regular Brownian motion with $\langle x^{2}\rangle ∼t$. This defines the free diffusion exponent $\gamma_{0}$. On the vertical axis, the exponent β characterizes resetting event frequency: a smaller β indicates fatter tails in the resetting time distribution and less frequent resetting; a larger β indicates thinner tails and more frequent resetting. As shown by the figure color code, in the β<1 region, regardless of α value, $\gamma =\gamma_{0}$. As β enters the $1<\beta <\gamma_{0}+1$ range, the diffusion exponent decreases linearly as $\gamma =\gamma_{0}+1-\beta$, until it reaches zero at $\beta =\gamma_{0}+1$. When $\beta >\gamma_{0}+1$, the diffusion exponent vanishes, corresponding to the MSD converging to a constant plateau (or logarithmic divergence for singular values α=1 and β=1).

The results presented herein demonstrate robustness against variations in the fundamental model outlined in Section 2. For instance, following Refs. [18,36], we examined the more realistic scenario of non-instantaneous stochastic resetting with ballistic return dynamics characterized by finite large speed. We found that short finite return times do not significantly modify the diffusion exponents γ, but slightly enhance the diffusion prefactors, $K^{\prime }$.

## Figures and Tables

**Table 1:** MSD for free Lévy walk (no SR): Numerical simulations versus theoretical predictions.

α	Theory	Simulation	Relative Error
0.25	$0.75\;t^{2}$	$0.74\;t^{2}$	1%
0.5	$0.5\;t^{2}$	$0.5\;t^{2}$	0%
0.75	$0.25\;t^{2}$	$0.26\;t^{2}$	4%
1	$1\;t^{2}/\ln t$	$1\;t^{2}/\ln t$	0%
1.1	$0.11\;t^{1.9}$	$0.13\;t^{1.9}$	18%
1.25	$0.3\;t^{1.75}$	$0.31\;t^{1.75}$	3%
1.4	$0.59\;t^{1.6}$	$0.56\;t^{1.6}$	5%
1.5	$0.89\;t^{1.5}$	$0.76\;t^{1.5}$	15%
1.6	$1.34\;t^{1.4}$	$1.08\;t^{1.4}$	19%
2	2 t	1.76 t	12%

## Data Availability

Data can be available upon reasonable request from all the authors.

## Abbreviations

The following abbreviations are used in this manuscript:

MSD	mean squared displacment
SR	stochastic resetting
CTRW	continuous-time random walk
